# Paralytic Shellfish Toxins in Mollusks from Galicia Analyzed by a Fast Refined AOAC 2005.06 Method: Toxicity, Toxin Profile, and Inter-Specific, Spatial, and Seasonal Variations

**DOI:** 10.3390/toxins16050230

**Published:** 2024-05-15

**Authors:** Juan Blanco, Juan Pablo Lamas, Fabiola Arévalo, Jorge Correa, Tamara Rodríguez-Cabo, Ángeles Moroño

**Affiliations:** 1 Centro de Investigacións Mariñas (CIMA), Xunta de Galicia, Vilanova de Arousa, 36620 Pontevedra, Spain; 2 Instituto Tecnolóxico para o Control do Medio Mariño de Galicia (Intecmar), Vilagarcía de Arousa, 36611 Pontevedra, Spain; plamas@intecmar.gal (J.P.L.); farevalo@intecmar.gal (F.A.); jcorrea@intecmar.gal (J.C.); tamara.rodriguez@edu.xunta.gal (T.R.-C.)

**Keywords:** saxitoxin, gonyautoxins, decarbamoyl STX, decarbamoyl GTX, biotransformation

## Abstract

Paralytic shellfish poisoning is an important concern for mollusk fisheries, aquaculture, and public health. In Galicia, NW Iberian Peninsula, such toxicity has been monitored for a long time using mouse bioassay. Therefore, little information exists about the precise toxin analogues and their possible transformations in diverse mollusk species and environments. After the change in the European PSP reference method, a refinement of the Lawrence method was developed, achieving a 75% reduction in chromatogram run time. Since the beginning of 2021, when this refinement Lawrence method was accredited under the norm UNE-EN ISO/IEC 17025, it has been used in the area to determine the toxin profiles and to estimate PSP toxicity in more than 4500 samples. In this study, we have summarized three years of monitoring results, including interspecific, seasonal, and geographical variability of PSP toxicity and toxin profile. PSP was detected in more than half of the samples analyzed (55%), but only 4.4% of the determinations were above the EU regulatory limit. GTX1,4 was the pair of STX analogs that produced the highest toxicities, but GTX2,3 was found in most samples, mainly due to the reduction of GTX1,4 but also by the higher sensitivity of the method for this pair of analogs. STX seems to be mainly a product of biotransformation from GTX2,3. The studied species (twelve bivalves and one gastropod) accumulated and transformed PSP toxins to a different extent, with most of them showing similar profiles except for *Spisula solida* and *Haliotis tuberculata*. Two seasonal peaks of toxicity were found: one in spring-early summer and another in autumn, with slightly different toxin profiles during outbreaks in relation to the toxicity during valleys. In general, both the total toxicity and toxin profiles of the southernmost locations were different from those in the northern part of the Atlantic coast and the Cantabrian Sea, but this general pattern is modified by the PSP history of some specific locations.

## 1. Introduction

Dinoflagellates are a group of algae that constitute an important part of the marine ecosystem. Among the dinoflagellate species, some have the capability to produce saxitoxin (STX) and other analog compounds. These compounds are alkaloids that block the sodium channels, at least in mammals, and can be accumulated by the organisms that feed on dinoflagellates, mostly in bivalve mollusks, that are filter-feeders. In marine environments, these toxins are known to be produced by several species of *Alexandrium*, *Gymnodinium catenatum*, and *Pyrodinium bahamense* [1]. The consumption by humans of bivalves that have accumulated high concentrations of these compounds, together with their biological activity, produces a syndrome known as Paralytic Shellfish Poisoning (PSP), characterized initially by numbness and tingling, usually starting in the lips and tongue but spreading to other parts after some time. With higher concentrations, total or partial paralysis of the extremities, together with other neurological and gastro-intestinal symptoms, may appear. In extreme cases, the intoxication can produce death by respiratory arrest [2,3]. PSP was discovered on the North American coast at the beginning of the 20th century, when an important number of intoxications were detected. The symptomology was traced back to 1793, during Captain Vancouver’s expedition to the North Pacific, when Mr. Menzies, a naturalist and surgeon, reported some intoxications, including a fatality, of members of the crew after eating mussels in Mathieson’s Channel, British Columbia [4]. After the development of a bioassay [5,6,7], paralytic shellfish toxicity was detected in many countries and oceans [8].

Bates and Rapoport [9] in 1975 found that saxitoxin could be oxidized to yield fluorescent derivatives, which allow for its detection by chemical methods. Several chromatographic methods were developed using: (1) ion exchange or ion pairing chromatography and subsequent oxidation of the eluates to detect the obtained peaks by fluorescence (post-column oxidation) [10,11,12,13]; or (2) direct separation and detection of the oxidation products by reverse phase chromatography and fluorescence (pre-column oxidation) [14,15]. However, in most countries, mouse bioassay was the reference method to be used in monitoring systems, and chemical methods were not generally used until 2017, when the Codex Alimentarius Commission classified method AOAC 959.08 (PSP mouse bioassay) as a Type IV method, suggesting that it cannot be used for monitoring, inspections, or regulatory purposes. Immediately after, the European Union (EU) set up in Regulation (UE) 2017/1980 [16] the method AOAC 2005.06 (HPLC pre-column) as the PSP reference method. Nowadays, the PSP European Reference Method is that described in Standard EN 14,526 (Commission Implementing Regulation (EU) 2021/1709) [17], which includes the HPLC pre-column method initially developed by Lawrence et al. [15], together with some posterior refinements and validations of other STX analogs whose validation was not initially included given the lack of some certified reference materials [18,19].

**Figure 1:**
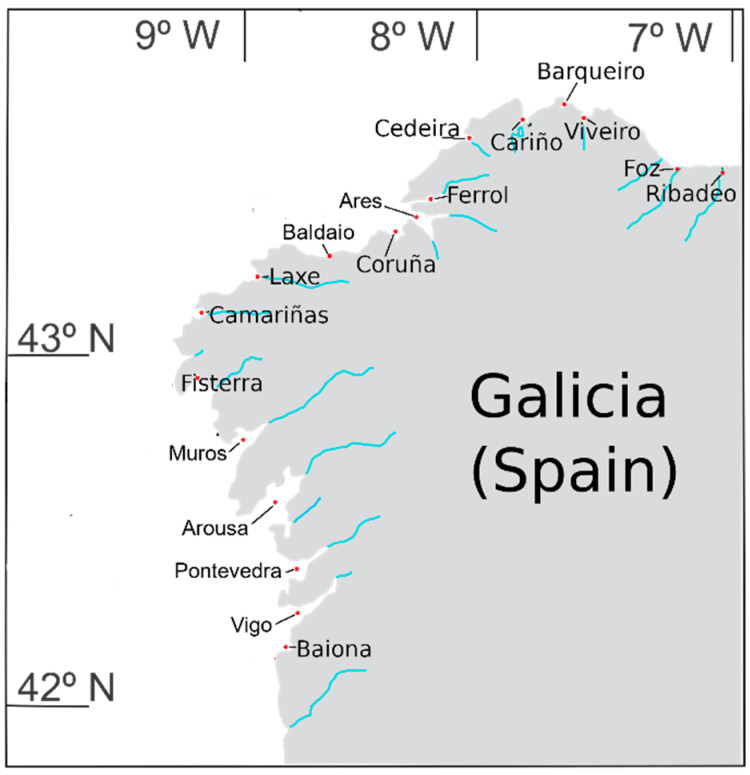
Geographical localization of the areas where the samples were collected (NW Spain).

Galicia (NW Spain) is an area of intense culture of mussels and exploitation of wild bivalve mollusk populations thanks to the system of coastal upwelling and circulation of the Rías, which drive high mussel growth rates [20]. To assure the safety for human consumption of this high bivalve mollusk production, a dynamical monitoring system was designed and implemented by the competent authorities [21]. Once the European Union decided to set up the method AOAC 2005.06 as the PSP reference method, it was clear that, in case of a PSP outbreak, due to its large sample process (extraction, cleanup, both periodic and peroxide oxidations, and fractioning), together with the necessity for full quantitative analysis of injecting four 15-min analyses per sample, it would only be possible to analyze a small number of samples per day, making it very difficult to get the results in 24 h due to the necessity of an extra day for interpretation of results [22]. Attempts have been made to refine the AOAC method, trying to achieve a reduction in chromatogram run time by using fused-core silica LC columns rather than fully porous particles [23,24] or by using the UPLC system [25]. Likewise, a still faster refinement of the AOAC 2005.06 method was developed at CIMA (Centro de Investigaciónes Mariñas) and Intecmar, which was implemented and accredited under the norm UNE-EN ISO/IOC 17,025 at Intecmar in January 2021. Since then, it has been used to carry out monitoring of PSP toxicity in Galicia. Previously, between 1995 and 2020, PSP monitoring was carried out using the method AOAC 959.08 with such toxicity detected and over the EU regulatory limit of 800 µg STX 2HCl equivalents·kg^−1^ in 6.5% and 1.6% of the analyzed samples, respectively [26]. The appearance of PSP toxicity in bivalves was linked to the presence of *Gymnodinium catenatum* or, much more frequently, to *Alexandrium minutum*. Both species have substantially different toxin profiles, with GTX4 and GTX1 being the predominant toxins in *A. minutum* [27,28,29] and several sulfocarbamoyl-toxins (mainly GTX5, GTX6, C1, and C2) in *G. catenatum* [30,31,32]. The toxins, once ingested by bivalves, undergo transformations that are toxin- and species-specific, which, consequently, modify the overall toxicity because the different toxin analogs have different toxic potencies (defined as toxicity equivalence factors, TEFs, relative to the toxicity of saxitoxin) [20]. The implementation of an HPLC-precolumn oxidation method in the monitoring of PSP toxins in Galician bivalves has allowed us to gather data on both the overall toxicities and toxin profiles. In this study, we have described the refinement of the AOAC 2005.06 method developed, the toxicities recorded in the area (Figure 1), the toxin profiles in different bivalve species and different locations, the seasonal variability on both toxicity and toxin profiles, and the relationships between toxin analogues during a period in which only *Alexandrium* blooms were detected.

## 2. Results

### 2.1. Refinement of the AOAC 2005.06 Method

**Figure 2:**
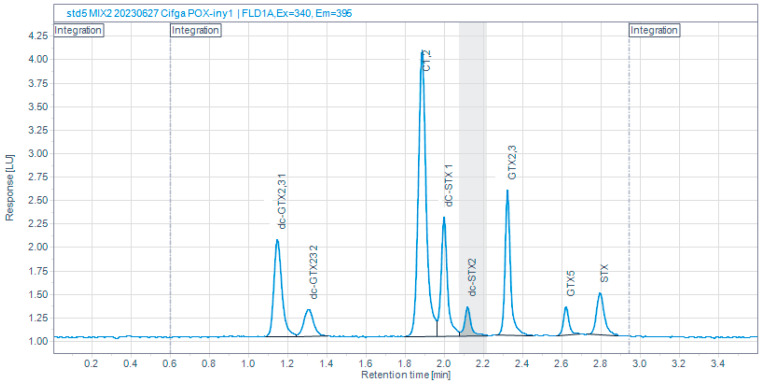
Chromatogram of a toxin mixture obtained with the refined (CIMA-Intecmar) method.

A 75% reduction in run time was achieved, from 15 to 3.6 min, by applying several refinements to the AOAC 2005.06 method, such as a different reverse phase column, a higher proportion of acetonitrile in mobile phase A, a higher volume of H_2_O_2_ for peroxide oxidation, filtering of mobile phases (Table 1), and a variation in the gradient program (Table 2). Despite this important reduction in runtime, it is still possible to get a resolution >1.5 between the two peaks of dcGTX2,3 and dcSTX in compliance with the method described in the norm EN 14526, which is the European PSP reference method (Figure 2).

### 2.2. Toxin Profile

**Figure 3:**
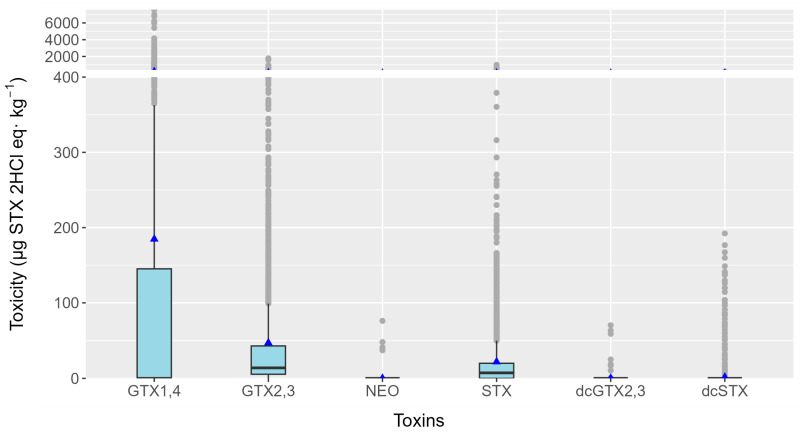
PSP toxicity estimated for each group of toxins detected. Boxes’ upper and lower bounds are the first and third quartiles, respectively. The horizontal line represents the median, triangles represent the arithmetic means, and dots represent outliers.

From January 2021 to October 2023, a total of 4567 mollusk samples were analyzed from 14 mollusk species (12 species plus raft and wild mussels). PSTs were found in 2511 samples, which constituted 55% of the total. Only 4.4% of the toxins found were above the legal limit (800 µg STX 2HCl equivalents·kg^−1^), which was about 8% of the samples where PST toxins were found. During the studied period, dcGTX2,3, dcSTX, GTX1,4, GTX2,3, NEO, and STX were detected (Figure 3), with GTX1,4 being the pair of analogs that, on average, contributed most to the toxicity of the bivalves (more than 3.9× the toxicity of the second toxin, GTX2,3, on average).

**Figure 4:**
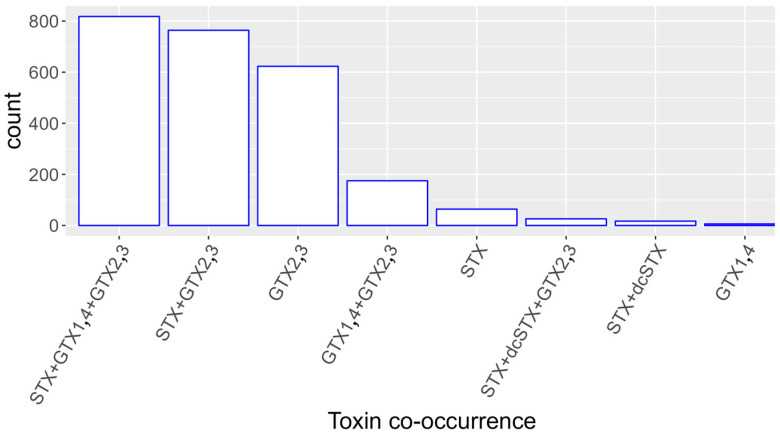
Co-occurrence of the studied toxins or groups of toxins in mollusk samples. Toxin combinations observed in less than five samples were not plotted.

GTX2,3 was the pair of toxins that appeared in most samples, mainly combined with STX and GTX1,4, but also alone (Figure 4). It seldom appeared in any other toxin combination. STX was usually found with GTX2,3 or with GTX2,3 and GTX1,4, but not with GTX1,4 in the absence of GTX2,3. GTX1,4 was nearly never found alone, being most frequently found together with GTX2,3 and STX. When zero was assigned to the values of GTX2,3 that were below the LQ of GTX1,4, the results were practically identical to those obtained with the original data.

### 2.3. Species-Specific Toxicities

**Figure 5:**
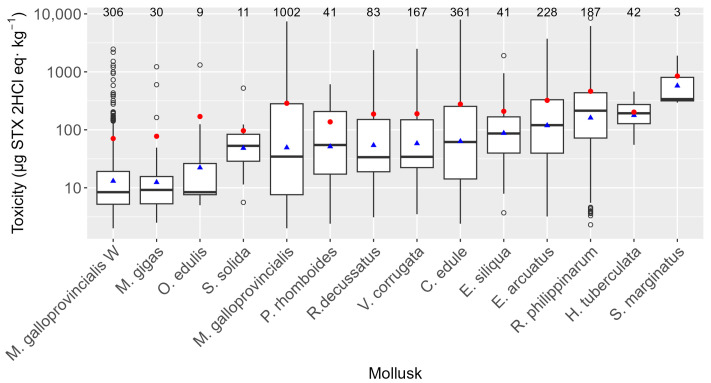
PSP toxicities recorded in the bivalve species studied. The upper and lower bounds of the boxes are the first and third quartiles, respectively; the horizontal line represents the median. Triangles represent the arithmetic means, and the shaded areas represent the distributions of the data. Dots represent outliers. The numbers of samples analyzed are shown at the top.

The average PSP toxicities were found to be different in the monitored species. Nevertheless, the Galician monitoring system (with various sampling frequencies and times depending on the evolution of PSP episodes) has an impact on the observed levels due to the following: (1) Cultured mussels (*Mytilus galloprovincialis*) are grown in rafts that are located far from the intertidal area (where most other species live), and, consequently, their levels cannot be directly compared with those of other bivalves. (2) Furthermore, for cultured mussels, in cases of simultaneous PSP and lipophilic toxins episodes with toxicity levels above the regulatory limit for lipophilic toxins, PSP is not newly analyzed until the first lipophilic result below the regulatory limit is obtained. (3) Wild mussels (*M. galloprovincialis* W), which in Galicia are not usually commercialized, are used as the sentinel species, being sampled until toxins are detected, in which case the commercial bivalve species start to be sampled instead of wild mussels. Taking all this into consideration, it was found that three razor clam species (*Solen marginatus*, *Ensis siliqua*, and *Ensis arcuatus*) and the Manila clam (*Ruditapes philippinarum*) were, on average (geometric mean), the most toxic bivalve species. Several commercially important species, such as the cockle *Cerastoderma edule*, the clams *Ruditapes decussatus*, *Venerupis corrugata*, *Polititapes rhomboides*, and *Spisula solida*, and the flat oyster *Ostrea edulis*, showed intermediate toxicity levels, while mussels *M. galloprovincialis* and the oyster *Magellana gigas* had moderate toxicities (Figure 5). The only gastropod sampled, *Haliotis tuberculata*, presented high average toxicity. Both maximum and minimum levels were detected in raft-cultured *M. galloprovincialis*.

**Figure 6:**
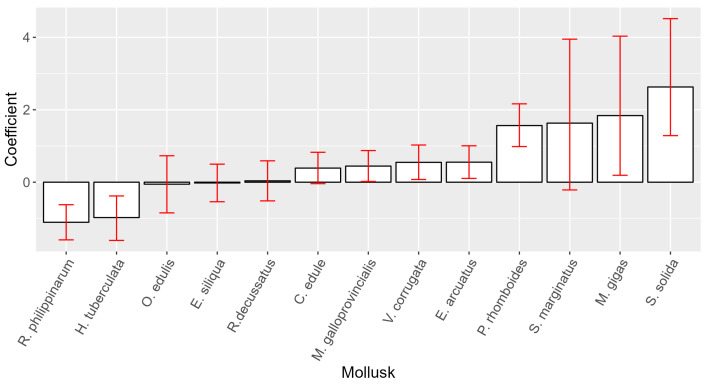
Bradley–Terry model coefficients with their 95% confidence intervals for the studied species. Wild *M. galloprovincialis* was used as a reference (coefficient = 0).

Attempting to rank the species studied by their capability to accumulate PSP toxins considering the peculiarities of the monitoring, we compared the concentrations of the different species by pairs when the samples were taken in the same week and from the same location (ría). We applied a Bradley–Terry model for paired preferences to that subset of data to obtain a coefficient for each species. The species with higher coefficients are those with a higher probability of reaching higher PSP toxicities. *S. solida*, *M. gigas*, *P. rhomboides*, and probably *S. marginatus* (with a wide confidence interval) seem to be the species that can accumulate more PSP toxicity. *R. philippinarum* and the gastropod *H. tuberculata* seem to be on the opposite end of the toxin accumulation scale. All other species had intermediate values, with coefficients only slightly different from those of mussels (Figure 6).

### 2.4. Species-Specific Toxin Profile

**Figure 7:**
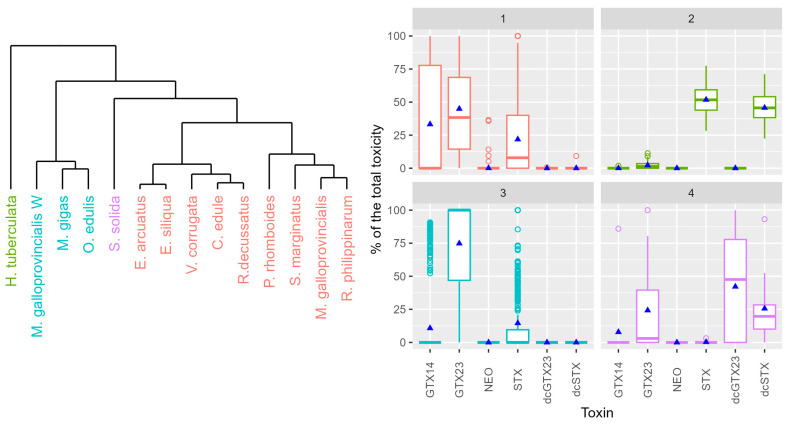
Cluster dendrogram of the species using the proportion of the toxins and the proportion of the toxins for each of the cluster groups (color). Upper and lower box limits are the first and third quartiles, the horizontal line inside each box represents the median, and triangles represent the mean.

By means of cluster analysis, four different profiles could be distinguished among the studied mollusk species (Figure 7). In the main group (group 1), GTX2,3 was dominant, but GTX1,4, and STX contributed noticeably (more than 20%) to the total toxicity. This group includes 9 out of the 14 species analyzed. The second group, which only includes the gastropod *H. tuberculata*, was characterized by the dominance of STX, with dcSTX as the second-largest contributor to the total toxicity and nearly no contribution of any other toxin. The third group was characterized by a clear dominance (more than 75%) of GTX2,3, with a low contribution of STX and nearly no contribution of other toxins. It includes *M. gigas*, *O. edulis*, and wild *M. galloprovincialis*, species that live on rocky substrates. Finally, the fourth group is characterized by a high contribution of two decarbamoylated toxins (dcGTX2,3 and dcSTX), but also of GTX2,3. Only one species, *S. solida*, is included in this group.

### 2.5. Seasonal Variation in Toxicity and Toxin Profile

**Figure 8:**
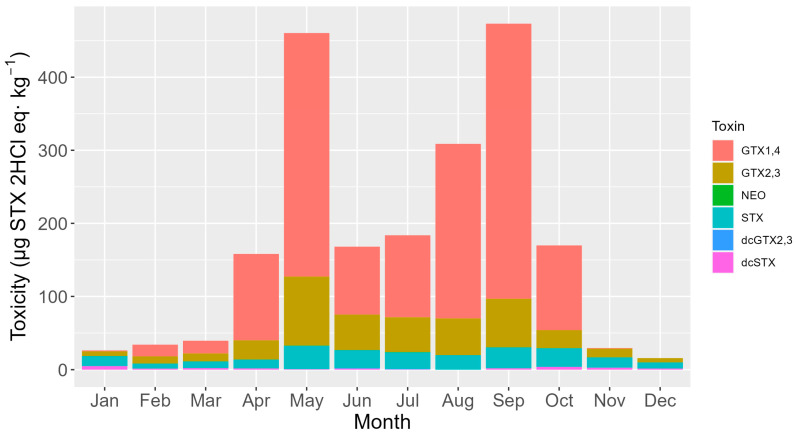
Seasonal variation of the average toxicity levels of the detected toxins.

**Figure 9:**
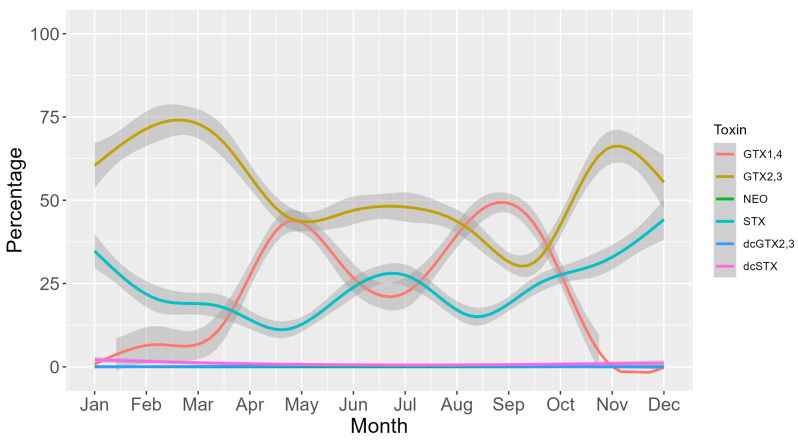
Seasonal variation in the percentage of each toxin. Lines are the loess-smoothed data, and gray areas are the corresponding 95% confidence intervals for the fitted curves.

The seasonal cycle of toxicity was characterized by two maxima, one in April-May and another in August–September, and two minima, the most pronounced of them during the winter months and the other one in mid-summer (Figure 8). During the two maxima, the toxicity rose substantially with the pair of analogs GTX1,4 (the toxins that mostly contribute to the total toxicity). This pair of toxins also exhibited a higher decrease right after the two maxima. GTX2,3 was the pair of toxins that mostly contributed to total toxicity in the mid-time between peaks (Figure 8). STX showed an annual trend like that of GTX2,3 but clearly increased its contribution during the winter months, this being the seasonal period in which GTX1,4 showed the lowest contribution (Figure 9).

### 2.6. Toxin Relationships over Time

**Figure 10:**
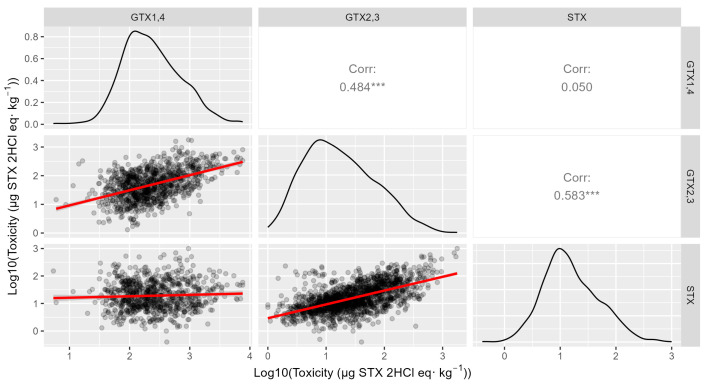
Relationship between the PSP toxicities estimated for each group of toxins using log-transformed data. The upper triangle shows the correlation coefficient corresponding to each pair, and the diagonal shows the distribution of the data. (*** indicates a probability level < 0.001).

The levels of the three main toxins were positively correlated between them, but those of STX were only reasonably well correlated with GTX2,3 (r = 0.58, log-transformed data) but not with GTX1,4 (r = 0.05, log-transformed data). All relationships seem to be approximately linear (Figure 10).

**Figure 11:**
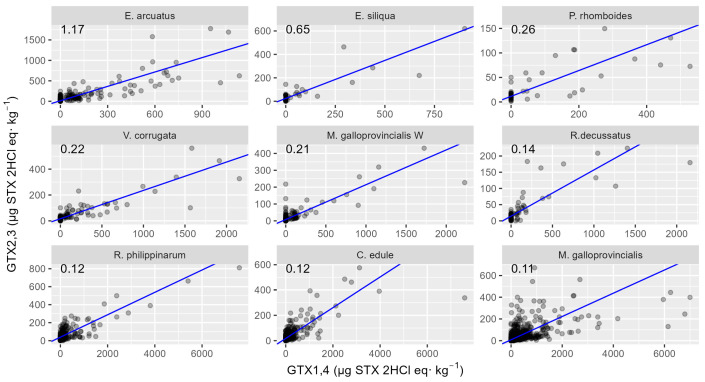
Relationship between GTX2,3 and GTX1,4 toxicities in the studied bivalve species, in which both toxins were detected in more than five samples. The numbers at the top are the slopes of the regression line.

**Figure 12:**
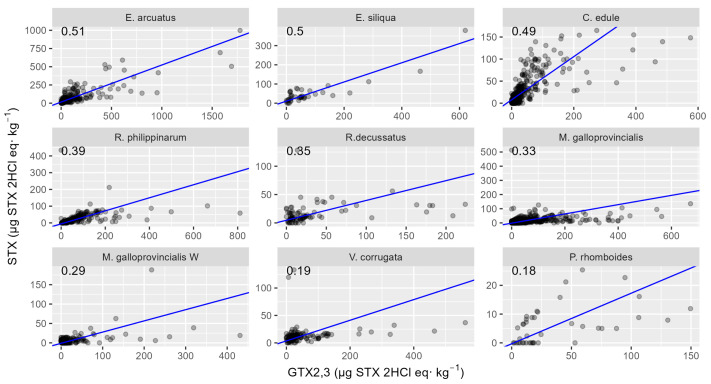
Relationship between STX and GTX2,3 toxicities in the studied bivalve species, in which both toxins were detected in more than five samples. The numbers at the top are the slopes of the regression line.

The relationship between toxin analogues changed as time passed since the start of each bloom, as might be expected from biotransformations. The regression analysis of GTX2,3 on GTX1,4 and the time elapsed from the start of the bloom show the effect of both independent variables to be positive and highly significant, therefore indicating that, as time passes after blooms, proportionally more GTX2,3 is observed in mollusk samples. The same was observed for the relationship between STX and GTX2,3. When GTX1,4 is added to the regression as an additional predictor, it has no significant effect, suggesting that only GTX2,3 and the time that has passed since the episode affect the STX concentration. The relationships between GTX2,3 and GTX1,4 and between STX and GTX2,3, which are expected to be determined by biotransformations, are species-specific (Figure 11 and Figure 12). For the relationship between GTX1,4 and GTX2,3, the differences between species are based on the capability of GTX1,4 reduction. Observing the linear relationship between GTX1,4 and GTX2,3, the two razor clam species, *E. arcuatus* and *E. siliqua*, showed the highest values of regression slope. Raft-cultured mussels showed the minimum slope value, with the cockle *C. edule* and two clams, *R. decussatus* and *R. philippinarum*, also showing low slopes. There was a noticeable difference between raft-cultured and wild mussels, with wild mussels having a higher slope than cultured ones.

The desulfation of GTX2,3 capability determines the differences between species in the relationship between STX and GTX2,3, and, as in the GTX2,3-GTX1,4 relationship, the two razor clam species, *E. arcuatus* and *E. siliqua*, showed the highest regression slopes. The way in which the other species were ranked was, however, very different. The cockle *C. edule* slope was very close to that of the razor clams, and *P. rhomboides* and *V. corrugata* had the lowest slopes. Contrarily to what was observed in the relationship GTX1,4-GTX2,3, in this case, no difference between wild and cultured mussels was observed, both presenting similar slopes.

### 2.7. Geographical Variation in Toxicity

**Figure 13:**
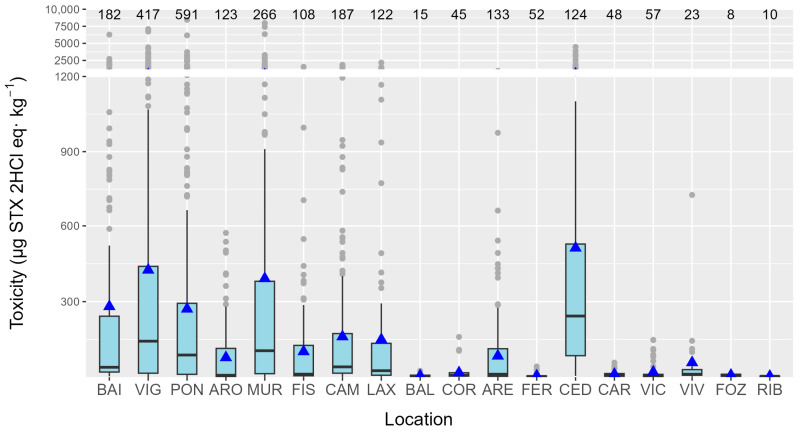
PSP toxicities recorded per ría along the Galician coast. The upper and lower bounds of the boxes are the first and third quartiles, respectively; the horizontal line represents the median. Triangles represent the arithmetic means, and the shaded areas represent the distributions of the data. Dots represent outliers. The numbers of analyzed samples in which at least one toxin was detected are shown at the top of the figure.

PSP toxicity levels were heterogeneously distributed along the Galician coast. Some locations had high PST levels, while the adjacent areas had low levels. The most affected locations were the rías of Cedeira, Muros, and Vigo, and the least affected were those in the Cantabrian Sea (Ribadeo, Foz, Viveiro, Vicedo, and Cariño), and Baldaio and Coruña on the Atlantic Coast (Figure 13).

## 3. Discussion

It is well known that the complete quantitative AOAC 2005.06 method is very time-consuming, limiting the number of samples that can be analyzed per day and delaying the generation of results. Two previous refinements of AOAC 2005.06 method chromatography had produced an approximately 50% reduction in chromatogram run time [24,25]. In this study, a 75% run time reduction was achieved, allowing us to analyze 20 samples per day even when, as in Galician PSP outbreaks, it is necessary to perform fractioning for GTX1,4 analyses. 

PSP toxins were detected in a high percentage of the samples obtained (55%). This prevalence is close to that computed from data obtained by the Portuguese monitoring system (42.7%) [33], where the Lawrence method has been routinely used for PSP analysis since 1996 [34]. In a previous study [26], using mouse bioassay results, we found a substantially lower PSP prevalence [26], as could be expected from the much lower sensitivity of the method used. The proportion of samples with PSP toxicity levels above the EU regulatory limit (incidence, 4.4%) was higher than it had been over the previous 25 years of mouse bioassay monitoring [26]. Relative to other toxins present in the area, this incidence is much lower than that of okadaic acid (10.1%) [35], which is the main lipophilic toxin in the area (DSP toxicity), but higher than that of domoic acid (ASP toxicity) [36]. The recorded incidence was similar to that in Portugal, even when the main responsible species are different [37], but it seems to be lower than in Scotland [38] (overall data are not given).

The toxin profile was characterized by the presence of three main groups of toxins, GTX1,4, GTX2,3, and STX, and by the much less frequent presence, limited to one or a few species, of dcGTX2,3, dcSTX, and NEO. GTX1,4 was the highest contributor to the recorded PSP toxicity. The observed toxin profile was as could be expected during a period in which only *Alexandrium* spp. was present, since, according to research on the *Alexandrium* isolates from Galicia, GTX4 predominates over GTX1, GTX2, and GTX3 [28,29]. The frequency of detection of the toxins, however, did not follow the same order as their contribution to the toxicity. GTX2,3 was the group that appeared in more samples, alone or combined with other toxins, mostly with STX but also in any other possible combination. This can be attributed to a combination of two factors. The first one is that the precolumn oxidation analytical methods are much less sensitive for GTX1,4 than for GTX2,3 and STX [13,23], and, therefore, the GTX2,3 group is more easily detected. However, practically the same results were obtained by deleting all GTX2,3 results that were below the quantification limit of GTX1,4. This factor, therefore, can be discarded as the main reason for the dominance of GTX2,3. The second factor is that, in most bivalves, GTX4 and GTX1 are reduced to GTX3 and GTX2 [39,40,41,42,43,44,45,46], and, consequently, the contribution of the first two toxins is expected to decrease relative to GTX2,3 as the depuration progresses. This transformation seems to be the main reason for the high frequency of GTX2,3, which is supported by a highly significant effect of the number of days that pass since the start of each episode on the regression of GTX2,3 on GTX1,4. Saxitoxin is not expected to be present in *Alexandrium*, but it could be generated by the desulfation of GTX2 [42,47,48]. As in the case of GTX2,3, the number of days since the start of each episode has a very significant effect on the regression of STX on GTX2,3. It does not seem likely that *G. catenatum* could have contributed to the toxins detected because this species mostly produces sulfocarbamoyl toxins [30,31] that were not detected in the studied period. The studied species did not accumulate PSP toxins to the same extent, nor did they show the same toxin profile. Notwithstanding, ranking the mollusk species by their average PSP toxicity is complex because of monitoring sampling peculiarities. Some species, such as wild mussels, are used only as sentinel species, and consequently, most samples are obtained when PSP levels are low or not detected. Additionally, some species are not present in all sampling locations. Consequently, mussels are among the species with the lowest average PSP levels, but they are also among those with the highest maximum levels. To try to rank the species in a more reliable way, we used paired comparisons, in which to compare pairs of species, only the determinations for one species that had a counterpart in other species taken from the same location at the same time were considered. Afterwards, the species were ranked using a Bradley–Terry model, which used wild mussels as the reference for comparisons. Based on this model, three groups of species could be roughly separated: (a) species with PSP levels similar to wild *M. galloprovincialis* (*O. edulis*, *E. siliqua*, *R. decussatus*, *C. edule*, raft-cultured *M. galloprovincialis*, *V. corrugata*, *and E. arcuatus*); (b) species with lower levels than wild *M. galloprovincialis* (*R. philippinarum* and *H. tuberculata*); and (c) species with levels higher than wild mussels (*P. rhomboides*, *M. gigas*, *S. solida*, and, with more uncertainty, *S. marginatus*). A previous study using mouse bioassay results [26] supports this species ranking, except for *P. rhomboides*, which did not show higher levels than mussels. Freitas et al. [49], in Aveiro, Portugal, found higher PSP levels in *S. marginatus* and *C. edule* than in *M. galloprovincialis*, roughly coinciding with our results. After a different bloom in the same area, however, *S. marginatus* and *C. edule* had, in general, lower PSP levels than *M. galloprovincialis* [46]. After a *Gymnodinium catenatum* bloom [50], similar levels of PSP toxins were observed in *C. edule* and *R. decussatus*, also supporting our results. Another mussel species, *M. edulis*, has been found to accumulate more PSP toxins than the Manila clam (*R. philippinarum*) [51,52,53] and the Pacific oyster (*M. gigas*) [51,52] in France and Korea, but attained similar toxicities in an experimental study in Japan [54], and in Poland, in a study that also included the cockle *C. edule*, obtaining the same result [55]. 

Toxin profiles were clearly species-specific. Two out of four groups obtained by cluster analysis were composed of only one species. One of them is the abalone *H. tuberculata*, which was characterized by the dominance of STX and dcSTX with practically no other toxin. Similar profiles have been found for this species in other samples from Galicia [56,57] and also in other *Haliotis* species from other geographical areas, such as Tasmania [58] or South Africa [59]. However, in abalones fed with kelp containing PSP toxins, the initial toxin profile was more complex, pointing to biotransformation as the main cause of the usual toxin profile [60]. In the same way, comparisons of toxin profiles in mussels, abalone viscera, and foot during a *G. catenatum* bloom revealed the possible biotransformations of PSP toxins in abalone foot, which was also the tissue with the lowest depuration rate [61]. The other one-species group, obtained by cluster analyses, included the clam *S. solida*, with a profile characterized by the high contribution of dcGTX23 and dcSTX. This species has been shown to have potent decarbamoylase activity [62,63], which explains this so-specific profile. The two other species groups (1 and 3), obtained by cluster analysis, present more comparable profiles with differences that appear to be the result of a more intense desulfation of GTX2,3 relative to a reduction of GTX1,4 in the organisms of the first group and the opposite in group 3. Surprisingly, those differences seem to be related to the habitat in which the species live more than to the species themselves, for two reasons: (1) the three species included in group 3 live on rocky shores, and those in group 1 live on sandy or muddy environments except raft-cultured mussels; and (2) wild and raft-cultured mussels are located in different groups even when they are the same species and their genetic information is very homogeneous [64]. 

Seasonality variation was characterized by two annual toxicity peaks, one in May and another in August–September. This pattern, presumably defined by *Alexandrium* blooms, is partially consistent with the one found in a previous study (25 years of mouse bioassay monitoring) [26], when the toxicity produced by *Gymnodinium catenatum* was not taken into consideration. The proportion of each toxin also varied with the season, and it was related to the total toxicity. Months when the peaks of toxicity were detected had proportionally higher GTX1,4 toxicity. This is consistent with the region’s known *Alexandrium* toxin profiles, which clearly show that GTX1 and GTX4 are dominant [27,28,29]. The very important effect of time passed since the episode began and the connections between the two groups of toxins support the idea that the profiles seen after the toxic peak are caused by GTX1,4 changing into GTX2,3 and then GTX2,3 changing into STX. In general, the nearly linear relationships found between GTX1,4, and GTX2,3 and between GTX2,3 and STX would imply that the uptake of the toxin from toxic cells could determine these relationships. However, the fact that there was no relationship between GTX1, 4, and STX, together with a curvature in the two previous relationships, suggests that transformations should be involved. As expected from the fact that GTX1,4 is reduced to GTX2,3 and GTX2,3 is desulfated to STX, the relationships between these two groups of toxins are not exactly linear. Instead, they have some curvature because the levels of the toxins that are the products of the transformations are higher than the levels of the toxins that are their parent molecules. The recorded differences in the relationships among the studied mollusk species, which share the source of toxic populations, also suggest that transformations play an important role in determining the toxin profile. The species seem to reduce or desulfate toxins at different rates, but the rates at which the two transformations take place seem to be independent. The two razor clams were the species that seemed to make the two transformations faster. The toxicity levels varied significantly across different locations, with a distinct disparity observed between the Cantabrian Sea and the Atlantic Coast. Generally, the Atlantic locations exhibited much higher levels of toxicity. The results obtained are roughly consistent with those found in the previous 25 years (with PSP monitored by mouse bioassay) [26], with relatively low levels of ría of Arousa (ARO) and high levels of Viveiro (VIV), Cedeira (CED), and Ares (ARE) in relation to the area in which they are located. 

## 4. Conclusions

A refinement of AOAC 2005.06 was carried out, achieving a 75% reduction in run time without compromising the required chromatographic resolution between toxins (>1.5). The prevalence of PSP toxins in Galicia was high (55%), but the percentage with toxicity levels above the EU regulatory limit was relatively low (4.4%), approximately five times higher than that of ASP and one-third that of lipophilic toxins. Three main groups of toxins (GTX1,4; GTX2,3; and STX) dominated the toxin profile, with GTX1,4 being the highest contributor to PSP toxicity. GTX2,3 was frequently detected, potentially due to method sensitivity and the transformation of toxins during depuration. Saxitoxin (STX) presence may be linked to the desulfation of GTX2,3. Bivalve species varied in PSP toxicity, with mussels having low average levels but high maximum levels. Ranking species by toxicity using paired comparisons suggests that *R. philippinarum* and *H. tuberculata* are the species that accumulate less toxicity than the others, and *S. solida*, *M. gigas*, *P. rhomboides*, and probably *S. marginatus* accumulate more than the others, with the remaining species having intermediate accumulation characteristics. Toxin profiles were species-specific, with the abalone *H. tuberculata* and *S. solida* showing a very distinct profile compared to the other species. Habitat seemed to influence toxin profiles, with rocky shore species differing from those living in sandy or muddy environments. Species-specific differences in relationships suggested varying rates of toxin reduction or desulfation. Seasonality showed two toxicity peaks (May and August–September), both related to *Alexandrium* blooms. Toxin proportions varied with the season, with higher GTX1,4 toxicity during peak months, suggesting toxin transformation during accumulation. There was significant variation in toxicity levels across locations, with the Atlantic Coast exhibiting higher toxicity than the Cantabrian Sea. The toxin profiles of the different locations seem to be affected by the total toxicity in the area, with GTX1,4 being a major contributor in high-toxicity locations.

## 5. Materials and Methods

### 5.1. Standards, Solvents, and Reagents

The Institute of Biotoxin Metrology at the National Research Council of Canada (NRCC, Halifax, NS, Canada) provided the certified reference materials saxitoxin (STX), neosaxitoxin (NEO), decarbamoylsaxitoxin (dcSTX), decarbamoylneosaxitoxin (dcNEO), gonyautoxin 5 (GTX5), gonyautoxin 6 (GTX6), gonyautoxins 1 and 4 (GTX1,4), gonyautoxins 2 and 3 (GTX2,3), decarbamoylgonyautoxins 2 and 3 (dcGTX2,3), and N-sulfocarbamoyl-gonyautoxins 2 and 3 (C1,2). All these toxins, plus gonyautoxin 6 (GTX6) and N-sulfocarbamoyl-gonyautoxins 1 and 4 (C3,4), were also acquired from Cifga S.A. (Lugo, Spain). The LC–MS-grade methanol was purchased from Honeywell (Charlotte, NC, USA). Acetonitrile LC–MS grade was purchased from Fisher Chemical (Madrid, Spain). Glacial acetic acid, ammonium acetate (96%), sodium chloride (99.5%), di-sodium hydrogen phosphate (99%), and sodium chloride were obtained from Merck (Darmstadt, Germany). Periodic acid (99.5%) and ammonium phosphate (98.1%) were from VWR (Radnor, PA, USA), and hydrogen peroxide solution (30%) and propan-2ol were obtained from Carlo Erba Reagents (Val de Reuil, France). Ultrapure water was obtained from a Milli-Q Gradient A-10 system (Millipore, Burlington, MA, USA). The reversed-phase Supelclean LC-18 (500 mg/3 mL) cartridges were acquired from Supelco (Bellefonte, PA, USA). Phenomenex (Torrance, CA, USA) supplied mixed-mode Strata-X-CW (60 mg/3 mL). Econofltr PES 0.22 µm syringe filters were purchased from Agilent Technologies (Santa Clara, CA, USA), and Nylon membrane filters, 0.22 µm, were from Filter-Lab (Sant Pere de Riudebitlles, Barcelona, Spain). 

### 5.2. Sampling

Samples were gathered on a weekly basis from all the production areas in Galicia where the harvesting of bivalve mollusks is allowed, as determined by the exploitation plans. The mussel, *Mytilus galloprovincialis*, whether cultured in rafts or from wild populations, was employed as a sentinel species. Upon the detection of a toxic event in mussels, further harvested species, including cockles, clams, oysters, razor clams, and queen scallops, were collected and analyzed. Wild mussels were not subjected to further analysis until the PSP episode concluded in certain instances. Harvesting wild mussels for human food is generally prohibited throughout Galicia, save in designated production areas.

There was uneven sampling of bivalve species, and several species were not sampled consistently over the entire year. The monitoring of raft mussels was particularly rigorous because of their usage as sentinels and the division of culture areas into numerous sub-areas, each of which was sampled separately. 

### 5.3. Sample Extraction and Preparation 

The AOAC 2005.06 [14] procedure for the extraction of PSTs was applied as follows: (1) double-extraction with 1% acetic ascid (first extraction at 100 °C), shaking for a few seconds, and centrifugation; (2) extract clean-up by SPE C18; and (3) pH adjustment to 6.5 using an automated pH-meter (Metrohm Hispania, Madrid, Spain). A filtered step using syringe filters was added to remove possible interfering particles in the oxidation reaction. The matrix modifier for periodate oxidation was prepared following the same extraction and clean-up protocol. Next, peroxide was used to oxidize the C18 extracts. The oxidation products of the non-N-hydroxylated toxins (dcGTX2,3; C1,2; dcSTX; GTX2,3; GTX5; and STX) were then analyzed. N-hydroxylated toxins (GTX1,4; NEO; dcNEO; and GTX6) were quantified in the fractions obtained by mixed-mode SPE cartridge fractionation [35] and oxidized using a periodate solution in the presence of matrix modifier. 

### 5.4. LC-FLD Determination Conditions

PSP toxins were determined using an Agilent Technologies (Wilmington, DE, USA) UHPLC-FLD system. The instrument consisted of an Agilent 1290 Infinity II Series LC system with a high-pressure binary pump, a multisampler (Agilent 1260 Infinity II) set at 4 °C, and an oven (Agilent 1290 Infinity II) for the LC column set at 40 °C. The fluorescence detector was an Agilent 1260 Infinity II using excitation and emission wavelengths of 340 nm and 395 nm, respectively. Chromatographic separation was performed in an Atlantis T3 column (75 mm × 2.1 mm, 3 µm) connected to an Atlantis T3 Vanguard cartridge (5 mm × 2.1 mm, 3 µm) (Waters, Milford, MA, USA). The mobile phase consisted of 0.1 M ammonium formate (A) and 0.1 M ammonium formate with 10% acetonitrile (B), both adjusted to pH 6 ± 0.1 with 0.1 M acetic acid, filtered through a nylon membrane filter, and sonicated for 15 min. The elution procedure was as follows: (1) a gradient program with a flow of 0.7 mL min−1 was run, starting at 0% B; (2) followed by a linear increment from 0 to 5% B (from minutes 0.0 to 0.3); an isocratic step with 5% B (from minutes 0.3 to 0.9); (3) after the isocratic step, a linear increment from 5 to 45% B (from minutes 0.9 to 2.1); (4) return linearity to 0% B at 2.5 min; and (5) in order to equilibrate the column, isocratic conditions at 0% B were held for 1.1 min. The total run was completed in 3.6 min. The injection volume was set at 15 µL (both for peroxide and periodate oxidations). OpenLab software v. 2.7 (Agilent) was used to acquire and process the chromatographic data.

### 5.5. Calibration and Toxicity Computation

Calibration curves with six toxicity points (computed from concentrations using the official EFSA TEFs [65] for each toxin) were run both at the beginning and at the end of each sequence of samples; the minimum acceptable correlation coefficient for both calibrations combined was 0.98. Calibration solutions had the following linear ranges in µg STX 2HCl equiv.L-1: 2.5–150 for dcGTX2,3, C1,2, dcSTX, GTX2,3, and STX; 0.3–15 for GTX5; 12–177 for GTX1,4 and Neo; 2.5–100 for dcNeo; 1.2–100 for GTX6 and 1.0–25 for C3,4.

PSP toxicity was estimated using the toxin concentrations measured by LC–FLD, and the toxicities of all toxins were added to give the total toxicity. For the toxins that cannot be individually analyzed (GTX1-GTX4 and GTX2-GTX3), the highest TEF of the two isomers was used.

Limits of quantification (LOQ) were experimentally determined by using a S/N ratio of 10:1. Furthermore, to meet the requirements of EN ISO/IEC 17025, recovery and precision for both repeatability and reproducibility were checked by spiking each toxin into different mollusk homogenates.

LOQ values in µg STX 2HCl equiv.kg^−1^ are: 40 for dcGTX2,3; 20 for C1,2, dcSTX, GTX2,3 and STX; 4 for GTX5; 144 for GTX1,4 and Neo; 60 for dcNeo, 18 for GTX6, and 101 for C3,4.

### 5.6. Statistical Analysis

The regression analyses were carried out using R [66] and the R package smatr 3 [67]. Plots were generated with the R package GGPLOT2 [68]. Ranking by pairs was carried out using the Bradley–Terry model with the R package BradleyTerry2 [69], and clustering was made with the R function hclust.

## Figures and Tables

**Table 1:** Summary of main parameter modifications carried out at CIMA-Intecmar to refine method AOAC 2005.06.

Parameter	AOAC 2005.06	CIMA-Intecmar
CH_3_CN mobile phase A	5%	10%
Mobile phases A and B	No filtering	Filter 0.22 µm Sonicate 15 min
Matrix modifier	Supernatant filtered to 0.45 µm	Filtered 0.22 µm
SPE-COOH cartridges	Bakerbone carboxylic acid silane	Strata-X-CW
Adjust pH oxidants	NaOH 0.2 M	NaOH 1 M
Volume of 10% H_2_O_2_ for peroxide oxidation	25 µL	50 µL
Mixing time after oxidation	Not specified	30 s
10% H_2_O_2_	Not filtered	Filtered 0.22 µm
Periodate oxidation reagent	Not filtered	Filtered 0.22 µm

**Table 2:** Chromatographic conditions and mobile phase gradient.

Column	Atlantis T3 C18, 3 µm, 2.1 × 75 mm		
Temperature	40 °C		
Flow	0.7 mL/min		
Injection volume	15 µL		
Autosampler temperature	4–6 °C		
Gradient	Time (min)	Phase A (%)	Phase B (%)
	0	100	0
	0.3	95	5
	0.9	95	5
	2.10	55	45
	2.50	100	0
	3.60	100	0

## Data Availability

Data are available under request from intecmar.gal.
